# The prospective usefulness of callous–unemotional traits and conduct disorder in predicting treatment engagement among detained girls

**DOI:** 10.1007/s00787-016-0869-7

**Published:** 2016-06-03

**Authors:** Olivier F. Colins, Lore Van Damme, Kostas A. Fanti, Henrik Andershed

**Affiliations:** 1 Department of Child and Adolescent Psychiatry, Curium-Leiden University Medical Center, Endegeesterstraatweg 27, 2342 AK Leiden, The Netherlands; 2 Department of Special Education, Ghent University, Ghent, Belgium; 3 Department of Psychology, University of Cyprus, Nicosia, Cyprus; 4 Center for Criminological and Psychosocial Research, Örebro University, Örebro, Sweden

**Keywords:** Conduct disorder, Treatment engagement, With limited prosocial emotions specifier, Antisocial behavior, Young offenders, Callous–unemotional traits

## Abstract

Although treatment engagement (TE) is crucial for treatment success it is not well known how likely detained girls are to engage in treatment and what features may impede them from doing so. This study is the first to examine the prognostic usefulness of two features of potential interest, being callous–unemotional (CU) traits and conduct disorder (CD), in relation to TE. Detained girls and their parents (*n* = 75) were interviewed with the Diagnostic Interview Schedule for Children to assess CD, and completed the Antisocial Process Screening Device to assess CU traits dimensionally and categorically as in the new diagnostic and statistical manual of mental disorders, fifth edition (DSM-5) CU-based specifier. One to two months later, the girls reported how much they engaged in treatment. At the zero-order level, self-, but not parent-reported CU traits and CD were predictive of lower levels of TE. The incorporation of CU traits into a diagnosis of CD identified girls with lower levels of future TE, a finding that held across different informants. Of note, the aforementioned findings only became apparent when using a dimensional measure of CU traits, and not when using the categorical measure of CU traits currently included in DSM-5. This study showed that CU traits can help developing an understanding of what factors hinder TE among detained girls. Our findings also support recommendations to incorporate CU traits into the CD diagnosis, and suggest that dimensional approaches to do so may yield relevant information about future levels of TE.

**Electronic supplementary material:**

The online version of this article (doi:10.1007/s00787-016-0869-7) contains supplementary material, which is available to authorized users.

## Introduction

Treatment engagement (TE) and its overlapping concepts treatment motivation and working alliance are considered to be a precondition for treatment planning [e.g., 35, 41], and have been positively associated with therapeutic change and successful clinical outcomes [e.g., 22, 33]. Because of this, TE is widely regarded as an essential initial outcome to be achieved and, thus, a critical step in the treatment process [e.g., 16, 18]. TE has typically been defined in a narrow way by focusing on behavioral indicators (e.g., treatment attendance). In contemporary conceptualizations, TE is defined as a multidimensional construct that not only includes observable behavior (e.g., collaboration with staff), but also attitudes (e.g., readiness to change), relational aspects (e.g., bonding with treatment staff), and cognitions (e.g., engagement in therapeutic activities, such as adopting problem-solving strategies) [18, 29].

## Treatment engagement among detained adolescents

Detained girls constitute a very troubled and vulnerable group of adolescents [49, 51] and are at risk for various undesirable outcomes in young adulthood [36, 54]. Notwithstanding that treatment services for these youngsters are urgently needed [50], TE is still understudied in detained adolescents. The limited amount of studies on the topic shows that low levels of TE are to be expected [27], especially among detained girls [18]. However, it is not well known what features or problems impede detained boys and girls, or some of them, from engaging in treatment. There is some evidence, though, to suggest that client characteristics that are highly prevalent in forensic samples can affect the level of TE in these criminal justice-involved adolescents. A recent study, for example, showed that detained girls with a conduct disorder (CD) diagnosis reported lower levels of TE than their counterparts without CD [53]. This finding converges with the idea that children and adolescents with CD are often distrustful and defiant to adults and, therefore, have difficulties to bond and collaborate with staff [e.g., 4]. A client characteristic other than CD that may affect the level of TE in detained adolescents is a callous–unemotional (CU) personality style. This CU style refers to a set of affective traits characterized by deficient empathy and guilt, insensitivity to others’ feelings, and shallow emotions. Detained adolescents with CU traits may be unconcerned about the consequences of their behavior and incapable to make strong attachments to others. These characteristics may affect the treatability of young people with CU traits, because they may be less likely to change or bond with treatment staff than youth without CU traits. However, these expectations have not yet been extensively tested, and were not supported by the only study on this topic among detained adolescents [48]. Clearly, more research on the relation between the client characteristics of CD and CU on the one hand, and levels of TE on the other hand, is warranted. For reasons mentioned below, studies on TE that incorporate CU traits into CD are particularly needed.

## Conduct disorder with callous–unemotional traits in relation to treatment engagement

Reflecting the growing body of evidence on CU traits [25], the diagnostic and statistical manual of mental disorders, fifth edition (DSM-5) added a CU-based specifier for the diagnosis of CD [2]. The specifier is labeled ‘with limited prosocial emotions (LPE),’ and is used when an individual meeting diagnostic criteria for CD exhibits two or more of the following criteria over at least 12 months and in multiple relationships and settings: (a) lack of remorse or guilt, (b) callous–lack of empathy, (c) shallow or deficient affect, and (d) unconcerned about performance [2]. Amongst other expectations, this LPE specifier is expected to support treatment planning for youths with CD [24, 34]. To date, no study has tested if CD individuals who met criteria for the LPE specifier as categorically defined by DSM-5 (CD + LPE) actually display lower levels of TE than individuals with CD who do not meet criteria for the LPE specifier (CD only).^1^ Filling this void is important, because evidence that CD + LPE girls are less likely to engage in treatment may further support the view that CD + LPE individuals require more intensive and tailored treatment interventions than their CD only counterparts [25]. Of note, prior empirical work predominantly incorporated dimensional measures of CU traits when studying children and adolescents with conduct problems. Therefore, studies on TE that uses dimensional measures of CU traits are relevant as well, especially because dimensional measures of CU traits may have stronger associations with constructs of interest (e.g., criminal behavior) than the categorically defined DSM-5 LPE specifier [30, 43].

## This study

This study aims to contribute to the scarce literature about TE in detained girls in various ways. First, despite the apparent increase in detained girls in recent years, detained girls still represent a vulnerable and understudied group within the criminal justice system [45]. This study will be the first to scrutinize the relationship between CU traits and CD in relation to TE in detained girls. Second, and notwithstanding that dimensional measures of CU traits have been applied when studying TE in detained boys [42], this study will also be the first to incorporate dimensional and categorical operationalizations of CU traits in detained adolescents with CD. Third, although parents of detained adolescents are difficult to locate, and often unwilling or unable to provide diagnostic information [e.g., 11], this study will also incorporate parent ratings of CU traits and CD. By doing so, this study is sensitive to recommendations to extend self-reports of CU traits with information from others [e.g., 2] and to assure that CD-TE and CU-TE associations, if any, are not solely explained by shared rater variance, being that only self-reported CD and CU predict self-reported TE [33, 39]. Fourth, through its short-term longitudinal design, this study will inform researchers and clinicians if self- and parent ratings of CD and CU traits collected shortly after detention entry provide information about future levels of TE. Fifth, as is the case in the US [15], detention facilities in Belgium provide various services to promote the girls’ rehabilitation, including education, (mental) health care, general support, and individual and group programs (e.g., social skills training). In this study, treatment, therefore, refers to any particular combination of group-based services (for all residents) and services tailored to the needs of each girl. Because well-circumscribed treatment programs are rarely available in detention facilities [12, 15], our broad definition of treatment increases the ecological validity of studying TE in detained girls and converges with prior work on TE that could not control for the type of treatment [48]. In addition, results from studies testing TE of youths being treated in highly controlled conditions are very difficult to generalize to real-world settings, where, for example, staff may be inexperienced, have had little training in managing treatment processes, and need to deal with multiple children [4, 22].

### Hypotheses

It was expected that both CD and CU traits would be negatively related to the levels of TE. In support of recent attempts to incorporate CU traits into a diagnosis of CD, it was also hypothesized that interaction effects between a CD diagnosis and CU traits in predicting levels of TE, would emerge. Based on prior work [30, 43], it was finally hypothesized that the strongest interaction effect between CU and TE would be revealed when using a dimensional measure of CU compared to using the categorically defined DSM-5 LPE specifier.

## Methods

### Participants

Participants included girls who were placed in an all-girl youth detention center in Flanders, Belgium, and one of their parents. Placement in this youth detention center is only possible following referral by a juvenile judge because of a criminal offense (e.g., shoplifting, burglary, and assault) or an urgent problematic educational situation (e.g., persistent truancy, running away, aggression, or prostitution), and is considered the harshest measure a juvenile judge can impose. Between February 2012 and June 2014, 169 girls were eligible to participate. Two girls could not be approached due to acute psychiatric crisis, 14 girls did not provide consent, and six parents refused their daughter’s participation, resulting in a participation rate of 87 % (*n* = 147). We also aimed to include one parent for each girl. A parent could participate if the following criteria were met: (1) sufficient contact with his/her daughter during the past year, varying from daily until at least monthly and (2) sufficient knowledge of Dutch. The latter criterion was based on the girl’s, staff’s, and interviewer’s assessment of the parent’s ability to participate in Dutch conversations and to read and comprehend the informed consent form. For the total sample of 147 girls, 115 girls had at least one parent meeting inclusion criteria. Fourteen girls did not provide informed assent to contact their parents. For 16 girls, the parents did not provide informed consent themselves, and for 10 parents who provided information, the girl’s self-reported TE was missing, resulting in a sample of 75 pairs of girls and one of their parents.

For the purpose of the present study, we will rely on 75 girls for whom TE data were available for at least one of both follow-up assessments during detention, and for whom parent ratings of CD and CU were available as well. The age of the girls (*n* = 75) ranged from 13.82 to 17.89 years (*M* = 16.22; SD = 1.13), 24 % was of non-Belgian origin, 52 % of a low socioeconomic status (SES) family, 29 % did not live with their parents, and 12 % had been detained in the past. These 75 girls were not significantly different from the girls who were not included in the present study (*n* = 72) regarding mean levels of self-reported CU traits, prevalence of self-reported CD, and socio-demographic features, with two exceptions: girls in this study (*n* = 75) were less often from an non-Belgian origin [24 versus 46 %, *χ* ^2^ = 7.73(1), *p* **<** 0.001], which might be due to the selection criteria, and had been detained less often in the past [12 versus 30 %, *χ* ^2^ = 6.66 (1), *p* **<** 0.05] than the girls who were not included (*n* = 72).

### Procedure

Participants were approached and assessed following a standardized protocol. The girls were addressed individually, receiving oral and written information about the aims, content, and duration of the study. The girls were assured that their information would be treated confidentially and that refusal to participate would not affect their judicial status or stay in the YDC. Written informed consent was given before starting the assessment. The girls’ parents also received a letter including information about the aims and practical aspects of the study and could refuse participation. CD and CU traits were assessed at the start of detention (i.e., within the first 3 weeks), and TE 1 and 2 months after the initial assessment. Participants were assessed in a private area in the YDC. The assessment was conducted by the second author or final-year university students, none of whom were on the staff of the YDC. Afterwards, the girls received oral and written information about the aim of contacting their parents/caretakers. After receiving the girl’s written informed assent to contact their parent/caretaker, an informed consent letter concerning their own participation was sent to these adults. The second author, then, tried to contact one parent/caretaker for each girl at least ten times over a 1-month period at varying times during the day, to check their willingness to cooperate and to make a telephone appointment at a time that suited the parent/caretaker the best. In most cases, the telephone assessment was conducted by the second author within 3 weeks after the girl had been assessed. Neither girls nor their parents received financial compensation. This study was approved by the Institutional Review Board of the Faculty of Psychology and Educational Sciences at Ghent University (2011/59) and by the Board of the YDC.

## Measures

### Outcome measure

#### Treatment engagement (TE)

TE was assessed by a 22-item self-report tool that was specifically designed for doing research with detained adolescents [9], was based upon two already existing tools [18, 29], and has been used in research with detained boys [9] and detained girls [53]. The 22 items were organized into four dimensions: readiness to change (five items; e.g., ‘I guess I have faults, but there’s nothing I really need to change’, ‘Maybe this place will be able to help me’; *α* in this study for the first/second assessment 0.74/0.83), bond with the staff (seven items, including two reverse scored items; e.g., ‘I trust the staff here’, ‘Staff here is genuinely concerned about my welfare’; *α* = 0.94/0.94), collaboration on goals and tasks (six items, including one reverse scored item; e.g., ‘I have established a good understanding with the staff here of the kind of changes that would be good for me’, ‘I am finally doing some work on my problems’; *α* = 0.76/0.78), and therapeutic engagement (four items; e.g., ‘I am willing to talk about my feelings during my stay here, ‘I have learned to analyze and plan ways to solve my problems’; *α* = 0.88/0.86). All 22 items were scored on a six-point rating scale, ranging from 0 (“not agree at all”; indicating low TE) to 6 (“definitely agree”; indicating high TE). Details about this TE tool can be retrieved in the online supplement.

For 11 of the 75 girls included in the present study, TE was only assessed at one of the two (most often the first) follow-up assessments. Therefore, we calculated the mean TE score for the girls with available TE data from both follow-up assessments, and for the remaining 11 girls, we used the TE score from the one assessment available to us. The correlation between the total TE score at the first TE assessment and the second TE assessment (*n* = 64) was 0.77 (*p* < 0.001), providing support for our approach. In addition, prior research using the same sample of detained girls did not reveal significant changes in TE between both follow-up assessments [53]. The inter-correlations between the four TE scale scores were 0.53^change-bond^, 0.61^change-collaboration^, 0.59^change-therapeutic^, 0.82^bond-collaboration^, 0.74^bond-therapeutic^, and 0.81^collaboration-therapeutic^ (all *p*s < 0.001).

### Baseline measures

#### Conduct disorder (CD)

The Diagnostic Interview Schedule for Children version IV (DISC-IV) is a highly structured diagnostic interview, designed for interviewing children 9–17 years of age, and can be administered by trained non-clinicians [47]. The Dutch DISC-IV child and parent versions were used to assess the past year prevalence of CD. The criteria of CD remained unchanged in the DSM-5, so the DISC-IV can be used to assess CD whilst referring to DSM-5.

#### Dimensional measure of callous–unemotional (CU) traits

CU traits were assessed by means of the self- and parent versions of the Antisocial Process Screening Device [APSD; 23]. The APSD consists of 20 items that tap psychopathic traits and are answered on a three-point rating scale: not at all true (0), sometimes true (1), or definitely true (2). Given the purpose of this study, only the CU factor (sum score of the six items) will be used. From here on, this dimensional measure of CU traits will be referred to as CU traits, unless otherwise specified. Cronbach’s alpha (*α*) for self- and parent-rated CU was 0.26 and 0.64, respectively. Because *α* penalizes short scales, we also calculated the mean-inter-item-correlation (MIC) that must be above the recommended cut-off of 0.15 [5]. The MIC for the self- and parent-reported CU dimension was 0.05 and 0.23, respectively. The low internal consistency for the self-report CU dimension of the APSD converges with prior work in criminal justice-involved youths [8, 44]. Because the APSD is currently among the most widely used measures to assess the LPE specifier, we, nevertheless, used its self-report CU dimension as well rather than just omit this from the analyses.

#### Categorical measure of CU traits: with limited prosocial emotions (LPE)

In line with all studies that used the APSD [e.g., 7, 30, 31, 40, 43, 52], girls were identified as meeting the LPE specifier criterion if they had a (reversed) score of 2 (definitely true) on at least two of the four APSD items that correspond to the LPE specifier criteria: item 12 (reverse scored) corresponds to criterion lack of remorse or guilt; item 18 (reverse scored) to criterion callous–lack of empathy; item 19 to criterion shallow or deficient affect; and item 3 (reverse scored) to criterion unconcerned about poor performance. From here on, this categorical measure of CU traits will be referred to as the LPE specifier. Once we only used the APSD self-report version, and once we only used the APSD parent version to identify girls who met criteria for the categorically defined LPE specifier. These variables will be referred to as self- and parent-reported LPE, respectively. The DSM-5 explicitly states that multiple information sources are necessary to assess the LPE specifier criteria, and, that self-reports must be extended with reports from others who have known the child for extended periods of time, including parents [2]. Therefore, we also identified girls who met criteria for the LPE based on self- or parent ratings. From here on, this variable will be referred to as OR-rule-based LPE.

#### Socio-demographics

Standardized information regarding age, origin, SES, family situation, and detention history was gathered by means of self-report. Girls were placed in the low (vs. moderate-to-high) SES category when both parents were unemployed or worked as (un)skilled laborers. Time between the baseline (CD and CU) and the first TE assessment was calculated, and is referred to as time in treatment.

## Data analyses

First, to test if CD, CU traits, and the LPE specifier were prospectively related to the four self-reported TE dimensions, regression analyses were performed and standardized beta coefficients (*β*) were calculated to examine the relationship between each predictor and the TE outcome variables. In these analyses, only one predictor at the time was included (see first hypothesis). Second, and for each of the four TE outcomes, regression analyses were performed that included the next sets of two predictors: (1) CD and CU traits and (2) CD and LPE. These analyses were required to test if CD remained predictive of TE after controlling for the shared variance with the dimensional or categorical measures of CU traits, and vice versa. Third, a multiplicative interaction term between the two predictors (e.g., CD × CU) was added to the aforementioned set of two predictors. When significant, change in *R* ^2^ will be reported in the running text (not in the tables) to show the additional variance explained by the interaction term. Dimensional variables were centered to facilitate the interpretation of the significant interaction terms, and the product interaction terms were computed from centered variables to reduce multicollinearity. For dichotomous variables, no centering is required. To probe the interaction effects, we used the procedures described by Aiken and West [1]. Details for all models, including the non-significant interaction terms, are available upon request. These interaction terms are relevant to test if the incorporation of CU traits in the diagnosis of CD provides information about future levels of TE (see hypotheses 2 and 3). To address issues of shared rater variance when studying TE [33, 39], all analyses were repeated whilst using parent-rated predictors (i.e., CD, LPE, and CU traits) and self-reported TE as outcome variables. Finally, for reasons mentioned earlier, the analyses were also repeated whilst using OR-rule-based predictors and self-reported TE as outcome variables. All analyses were two-tailed with *p* < 0.05 as an indication for statistical significance. Data analyses were performed using SPSS 23.0.

## Results

### Descriptive statistics

Descriptive information in terms of the number of girls with CD, with the LPE specifier, and with CD + LPE, and in terms of mean level scores for CU traits and TE, is presented in Table 1. Time in Treatment (*M*_days_ = 31.81; SD = 4.27) was not significantly related to any of the four TE scales, and, therefore, will not be included in the regression analyses as a control variable.

**Table 1:** Descriptive information (*n* = 75)

Categorical predictors	*N* ^a^	%		
Self-reported CD	36	48.0		
Self-reported LPE	13	17.3		
Self-reported CD + LPE	10	13.3		
Parent-reported CD	37	49.3		
Parent-reported LPE	42	56.0		
Parent-reported CD + LPE	27	36.0		
OR-rule-based CD	55	73.3		
OR-rule-based LPE	45	60.0		
OR-rule-based CD + LPE	38	50.7		

Dimensional predictors	*M*	SD	Range	Skewn.
Self-reported CU score	4.21	1.76	0–8	−0.05
Parent-reported CU score	7.28	3.03	0–12	−0.33

Outcomes	*M*	SD	Range	Skewn.
Readiness to change	15.51	6.39	1–28	−0.08
Bond with staff	20.62	10.02	0–38	−0.22
Collaboration on goals	20.32	7.18	0–36	−0.22
Therapeutic engagement	12.52	5.89	0–24	−0.10

*CD* conduct disorder, *LPE* limited prosocial emotions, *CU* callous–unemotional

^a^ *N* refers to the number of girls on a total of 75 participants

### Self-reported CD and CU traits as predictors of self-reported treatment engagement

#### Self-reported categorical measure of CU

At the zero-order level (Table 2, Models 1), CD was significantly negatively related to bond and collaboration, whereas the LPE specifier was not significantly related to any of the four TE dimensions. After controlling for the shared variance of CD and the LPE specifier, CD remained significantly negatively related to bond (Table 2, Model 2). There was no significant interaction between the CD and the LPE specifier in predicting TE outcomes.

**Table 2:** Self-reported conduct disorder (CD) and callous–unemotional (CU) traits in relation to self-reported treatment engagement (*n* = 75)

	Categorical measure of CU traits				Dimensional measure of CU traits			
	Change	Bond	Collab.	Therap.	Change	Bond	Collab.	Therap.
Models 1	*β*							
CD^a^	−0.13	−**0.31**	−**0.26**	−0.21	−0.13	−**0.31**	−**0.26**	**−**0.21
CU traits	−0.19	−0.20	−0.20	−0.21	−**0.36**	−**0.41**	−**0.45**	−**0.40**
Model 2								
CD	−0.08	−**0.28**	−0.22	−0.16	−0.05	**−0.23**	−0.17	−0.13
CU traits	0.16	−0.13	−0.13	−0.16	−**0.35**	−**0.35**	−**0.41**	−**0.37**

Bolded betas significant at *p* < 0.05; time in treatment was not significantly related to the TE scales, and was not included as control variable

*Models 1* one predictor at once, *Model 2* two predictors at once, *Beta* standardized beta coefficients, *Collab.* collaboration, *Therap.* therapeutic engagement

^a^The standardized betas for columns 2–5 (Categorical measure of CU traits) are identical to the standardized betas for columns 6–9 (Dimensional measure of CU traits). For clarity and ease of interpretation, we present them twice

#### Self-reported dimensional measure of CU

At the zero-order level (Table 2, Models 1), CD was significantly negatively related to bond and collaboration, whereas CU traits were significantly negatively related to all four TE dimensions. After controlling for the shared variance of CD and CU traits, CD remained significantly negatively related to bond, whilst CU traits remained significantly negatively related to all TE outcomes, being change, bond, collaboration, and therapeutic engagement (Table 2, Model 2). Of note, the interaction between CD and CU in predicting therapeutic engagement was significant (*β* = −0.39; Δ*R* ^2^ = 0.06; *p* = 0.02), while the main effects were non-significant (CD: *β* = −0.13, *p* = 0.23; CU: *β* = −0.06, *p* = 0.71). Additional analyses showed that the CU was significantly negatively related to therapeutic engagement in girls with CD (*β* = −0.57; *p* < 0.001), but not in girls without CD (*β* = −0.06; *p* = 0.70).

### Parent-reported CD and CU traits as predictors of self-reported treatment engagement

#### Parent-reported categorical measure of CU

CD and the LPE specifier were not related to TE dimensions at the zero-order level (Table 3, Models 1) or after controlling for their overlap (Table 3, Model 2). No significant interaction effects emerged between the CD and the LPE specifier in predicting TE.

**Table 3:** Parent-reported conduct disorder (CD) and callous–unemotional (CU) traits in relation to self-reported treatment engagement (*n* = 75)

	Categorical measure of CU traits				Dimensional measure of CU traits			
	Change	Bond	Collab.	Therap.	Change	Bond	Collab.	Therap.
Models 1	*β*							
CD^a^	0.12	0.02	−0.01	0.02	0.12	0.02	−0.01	0.02
CU traits	−0.01	−0.04	−0.08	−0.20	−0.02	−0.11	−0.15	−0.17
Model 2								
CD	0.16	0.04	0.02	0.10	0.16	0.10	0.09	0.13
CU traits	−0.13	−0.05	−0.11	−0.23	−0.09	−0.14	−0.19	−0.23

Bolded betas significant at *p* < 0.05; time in treatment was not significantly related to the TE scales, and was not included as control variable

*Models 1* one predictor at once, *Model 2* two predictors at once, *Beta* standardized beta coefficients, *Collab.* collaboration, *Therap.* therapeutic engagement

^a^The standardized betas for columns 2–5 (Categorical measure of CU traits) are identical to the standardized betas for columns 6–9 (Dimensional measure of CU traits). For clarity and ease of interpretation, we present them twice

#### Parent-reported dimensional measure of CU

CD and CU were not related to TE dimensions at the zero-order level (Table 3, Models 1) or after controlling for their shared variance (Table 3, Model 2). Of note, the interaction between CD and CU in predicting therapeutic engagement was significant (*β* = −0.33; Δ*R* ^2^ = 0.06; *p* = 0.039), while the main effects were non-significant (CD: *β* = −0.16, *p* = 0.21; CU: *β* = −0.02, *p* = 0.88). Additional analyses showed that the CU was significantly negatively related to therapeutic engagement in girls with CD (*β* = −0.44; *p* < 0.01), but not in girls without CD (*β* = −0.02; *p* = 0.89).

### OR-rule-based CD and LPE as predictors of self-reported treatment engagement

At the zero-order level, OR-rule-based CD was negatively related to bond (*β* = −0.26; *p* = 0.022) and to collaboration (*β* = −0.24; *p* = 0.035), two findings that remained significant after controlling for the shared variance with the OR-rule-based LPE specifier (*β* = −0.27; *p* < 0.025 and *β* = −0.25; *p* < 0.048, respectively). No other significant relations emerged at the zero-order level and none of these relations remained or became significant after controlling for the shared variance between the OR-rule-based CD and the LPE specifier. No significant interaction effects emerged between the OR-rule-based CD and the OR-rule-based LPE specifier in predicting TE, although the interaction with therapeutic engagement approached significance (*β* = −0.53; *p* = 0.053). Additional analyses, nevertheless, showed that the OR-rule-based LPE specifier was neither significantly related to therapeutic engagement in girls with CD (*β* = −0.25; *p* = 0.065) nor in girls without CD (*β* = 0.27; *p* = 0.25).

## Discussion

Although treatment engagement (TE) is likely to be crucial for treatment success, it is not well known how likely detained girls are to engage in treatment and what features may impede them from doing so. This study is the first to examine the prognostic usefulness of two features of potential interest, being CU traits and CD, in relation to TE. As such, the study’s findings should be interpreted with caution until future work replicates these results.

### Conduct disorder and treatment engagement

It has been argued that bonding with staff and collaborating on goals may be particularly difficult for conduct disordered individuals, for example, because they are often defiant to and distrustful of adults, and blaming others for their problems [4]. Interestingly, the present study supported this link by revealing a negative association between a diagnosis of CD and the TE dimensions bond with staff and collaboration on tasks and goals. Of note, these relations could only be revealed when relying on self-reports of CD (Table 2), and not when relying on parent reports (Table 3). From a methodological point of view, this finding might seem unfortunate, because it suggests that shared rater variance may drive the relation between self-reported CD and self-reported TE. From a clinical point of view, this finding might nevertheless be welcomed because relatively easy to get information (self-reports) may help to identify detained girls who are unlikely to engage in treatment. In addition, our findings support the suggestion that the validity of parent-report measures on personality and antisocial behavior decreases in adolescence, whereas the validity of self-report measures increases during adolescence [32].

### Dimensional and categorical measures of CU traits and treatment engagement

Being too DSM CD centric may limit our understanding of the potential role of the new LPE specifier in designating a distinct subgroup of antisocial adolescents with serious conduct problems that may not meet criteria for CD [26]. Therefore, research that uses the LPE specifier by itself, that is, without linking it to a CD diagnosis is warranted as well. The models (Models 1) in this study that included CU traits as sole predictor, indeed, showed that dimensionally measured CU traits were negatively related to readiness to change, bond with staff, collaboration on tasks and goals, and therapeutic engagement, though only when relying on self-report. Importantly, for all four TE dimensions, these relations remained significant after controlling for the overlap between CU and CD. This suggests that there is something unique in dimensionally assessed CU traits that is not captured by a CD diagnosis, thereby underscoring the relevance to study CU traits in a DSM non-CD centric manner as well. Of note, parent ratings, again, were not significantly related to TE dimensions, suggesting that self-ratings are useful for prognostic purposes, despite the very low internal consistency of the CU scale used in this study.

### Incorporating CU traits into a diagnosis of CD in relation to treatment engagement

The scarce but available work in detained boys and girls showed that DSM-5 LPE specifier is of restricted usefulness to identify CD youths who differ from other CD youths in terms of criminal behavior, aggression, and psychiatric morbidity [7, 10, 13, 52]. This new categorically defined specifier also is expected to be relevant for treatment planning [25]. Though only one facet of the treatment process, studying whether CD + LPE detained girls are less likely to engage in treatment than CD only girls, is a first step in empirically testing this expectation. This study’s finding, however, did not reveal any significant interaction between a diagnosis of CD and the LPE specifier, suggesting that this categorically defined DSM-5 specifier does not differentiate between girls with CD who vary in levels of TE. As this is only the first study on the topic, future research is warranted before firm conclusions can be drawn. Interestingly, when using self-report or parenting ratings, a significant interaction effect emerged between CD and dimensionally measured CU traits in predicting therapeutic engagement. This finding not only converges with our hypothesis that dimensional measures of CU traits will be more useful than the categorically defined LPE specifier, but also emphasizes the importance of further testing alternative strategies to incorporate CU traits into DSM-5 CD.

### Strengths and limitations

Strengths of the present study include the focus on detained girls, the neglected gender in this kind of research, the longitudinal design, and the inclusion of a substantial number of parent reports. Although the number of parents included may seem rather low, researchers often have great difficulties to include parents from detained youths, which explain why few studies manage to include a substantial percentage of parents [e.g., 35 out of 160; 21].

Our findings also must be interpreted in the context of some limitations. First, TE was assessed by self-ratings only, and therefore, we were not able to test if the findings would be replicated when using detention staff ratings. A prior study, nevertheless, showed that self-reported CU traits were not related to staff-rated TE [48]. Second, the girls who were not included in the study were more often detained in the past than the girls that were included in this study. Even though there were no significant differences between both groups in mean levels of CU traits and in the prevalence of CD, it is likely that these excluded girls were the ones with the lowest TE levels, a possibility that underscores the potential relevance of including staff ratings of TE and antisocial behavior (e.g., institutional misdemeanor). Third, the TE assessment was restricted to 1 and 2 months after baseline. Therefore, it can be argued that future studies should explore whether the prospective relations between TE and variables of interest remained when using longer follow-up periods. However, in the facility, where the study’s participants were recruited, girls are on average detained for 3 months. As such, our restricted time frame increases the ecological validity of the findings. Fourth, our TE measure is new and has not yet been established as well-validated and reliable. Therefore, our results need to be interpreted with caution, especially since the correlation between some of the TE dimensions was very high in the present study, suggesting that some scales substantially measure the same construct. Yet, high inter-correlations between TE scales have been reported in prior work with detained adolescents that relied on other tools to gather self-ratings of TE (e.g., *r*’s between 0.84 and 0.87 [48]). If this overlap between scales consistently emerges in future work on TE, it will be relevant to keep refining assessment tools designed to measure the multidimensional construct TE is considered to be. Fifth, it is well established that there is substantial individual variability in the rate of change of CD [e.g., 38] and CU traits over time [e.g., 20], and in children being identified to meet criteria of CD and LPE over the years [43]. Thus, our one-time point assessment of CU and CD can be considered to constitute a limitation that should be addressed in future work, though it must be mentioned that decreasing CU groups has not always been found [38]. Finally, due to sample size, we were not able to include various other predictors of interest. As a consequence, we could, for example, not test if CU traits in combination with other personality traits (e.g., grandiose-deceitfulness) were even more predictive of TE [46]. Yet, the focus of the present study was on the incorporation of CU traits into a CD diagnosis. Given recent proposals to include the other psychopathic traits in DSM-5-based CD [46], future work should investigate which subtyping approach works best: only incorporating CU traits or including the entire psychopathy construct.

### Implications

Youth with (versus without) CU traits have shown poorer responses to treatment, lower rates of treatment participation, and lower rated quality of participation [e.g., 19, 28, 42]. The present study suggests that these results can be explained by low levels of TE seen in youth with high levels of CU, especially since TE has been considered crucial for treatment success. The time detained girls are available for treatment is often restricted. Therefore, the sooner clinicians are able to identify girls who are particularly unlikely to engage in treatment, the sooner they can start with seeking ways to increase levels of TE, for example, using motivational approaches and techniques [3]. This study provides a novel evidence that self-rating of CD and CU traits may help to identify a small group of girls who are particularly resistant to engage in treatment. Even though these relations can partially be explained by shared rater variance, the evidence that brief self-ratings of CU traits are able to do this is particularly relevant for juvenile detention staff that often lacks time to perform comprehensive assessment for every girl entering the facility [14]. Moreover, the finding that parent ratings of CU traits were only predictive of one of the four TE dimensions suggests that parents might not add much information when the goal is to inform clinicians about future levels of TE as reported by the girls themselves. This finding too is particularly interesting for detention personnel, because parents are not easy to locate, or often unwilling or unable to provide information that serves assessment purposes. Importantly, the present study does not allow concluding that parent ratings on features other than CU traits and CD do not predict future levels of TE as reported by the girls, or that parents can actually be relevant raters of their girls’ TE themselves.

Our findings should not be overstated, since the additional variance in levels of TE explained by levels of CU traits was small when not simultaneously ascertain if girls do or do not have CD (16 % at best). Yet, the likelihood to make long-lasting changes in a short period is also small, so it would already be a success if one could increase these girls’ TE to such an extent that they are at least willing to engage in treatment after being released into the community and referred to less restrictive treatment facilities. If detention staff do have time to ascertain whether girls meet criteria for CD, our findings showed that dimensionally measured CU traits explained 25 % (parent-rated CU) and 36 % (self-rated CU) of the variance in levels of the TE dimension therapeutic engagement. This later TE dimension includes items that ask the girls if they have learned to analyze and plan ways to solve their problems, and if they followed detention staff’s guidance. Thus, it can be speculated that CD girls with higher levels of CU traits are most likely unable or unwilling to profit from the treatment being provided. Our data do not provide evidence to support this latter speculation. Therefore, future work on treatment outcomes is warranted, especially because it is still possible that detained girls with CU traits may benefit from treatment even if treatment outcomes remain worse when compared to youth without CU traits.

Categorical thinking persists in clinical practice [6], and clinicians, thus, may find it more easy to use the LPE specifier (with versus without CU traits) than using a dimensional score of CU. Unfortunately, all support for a link between CU traits and TE stemming from the present study could not be replicated when using the categorical measure of CU traits being included in DSM-5. Thus, it remains an open question how clinicians can or will use the evidence provided here in their work with detained girls. However, before they consider doing so, research is warranted that tests the link between CU traits and TE as part of a clinical protocol where the information may have real consequences for the informant rather than as part of a research protocol where anonymity and confidentiality of the informant and information are guaranteed, as in the current study.

## Electronic supplementary material

Below is the link to the electronic supplementary material.

Supplementary material 1 (DOCX 15 kb)

## Footnotes

1 In 41 clinic-referred boys with conduct problems (*M*_age_ = 9.60), boys with elevated (versus low) levels of CU traits were rated by their clinicians as less responsive to the idea of changing their behavior [17]. However, only 6 boys in this study met criteria for CD, which implies that these findings may not extend to boys with conduct problems that are severe enough to receive a CD diagnosis, and that a categorical approach other than the one used in DSM-5 was used to differentiate between boys with high and low levels of CU traits. Thus, research on the concrete incorporation of CU traits into CD in relation to TE is lacking.
