# Supplementary material for: The prospective usefulness of callous–unemotional traits and conduct disorder in predicting treatment engagement among detained girls
Source: Eur Child Adolesc Psychiatry. 2016 Jun 3;26(1):75–85. doi: 10.1007/s00787-016-0869-7 (PMC5233744; doi:10.1007/s00787-016-0869-7)
Supplement: Supplementary file 1 — Supplementary material 1 (DOCX 15 kb) [file 787_2016_869_MOESM1_ESM.docx]

Online Supplementary Material

**Treatment Engagement Measure**

At the start of this study and prior work on TE in detained boys and girls [9], there was no self-report tool available that assessed TE in a way that accounts for the specific setting of detention facilities (see Introduction). To fill this void, researchers [9] first combined and modified a 17-item self-report tool that was designed to assess *Readiness to Change, Bond With the Staff,* and *Collaboration on Goals and Tasks* [18] with four self-report items that focus on *Therapeutic Engagement* [26]. Second, to make these 21 items applicable for doing research with detained youths, the word “staff” from the original 17-item tool was replaced by a Dutch word (‘groepsleiders’) that refer to the professionals who monitor, supervise and act with these youths in various activities, including cooking, cleaning, free-play, individual assignments, group discussions, and social skill training. Likewise, the word ‘counseling’ from the original 4-item scale was replaced with ‘my stay here’. Third, to easy readability and to be consistent across items, several items were rephrased into the active voice, and one item was added to the dimension *Collaboration on Goals and Tasks*.
